# COVID-19 related information seeking: The impact of media on parental concerns

**DOI:** 10.3389/fpubh.2022.977634

**Published:** 2022-10-13

**Authors:** Yann Arnaud, Olivier Drouin, Roxane Borgès Da Silva

**Affiliations:** ^1^Center for Interuniversity Research and Analysis on Organizations (CIRANO), Montreal, QC, Canada; ^2^Division of General Pediatrics, Department of Pediatrics, CHU Sainte-Justine, Montreal, QC, Canada; ^3^Department of Social and Preventive Medicine, School of Public Health, University of Montreal, Montreal, QC, Canada; ^4^Department of Management, Evaluation and Health Policy, School of Public Health, University of Montreal, Montreal, QC, Canada

**Keywords:** COVID-19, parental concern, information sources, media use, cluster analysis

## Abstract

The expansion of information sources and their use has accelerated since the beginning of the COVID-19 pandemic, sometimes provoking significant concern in the daily lives of parents. The objective of this study was to investigate the association between COVID-19 related information sources and the level of concern about COVID-19 among parents of school-aged children. Using factor analysis and hierarchical ascending classification, we constructed groups according to the information sources they used. We performed ANOVA analysis and then binomial logistic regression to compare concern levels among the groups created. Overall, the 3,459 participants were mainly women (79.2%) and 59.5% reported being between 35 and 44 years old. The mean concern score in our sample was 9.5/15 (s.d. = 3.87). The whole sample fell into three groups: (1) Traditional Media (*n* = 1,610), who mainly used newspapers; (2) Online Social Networks and Entourage (*n* = 776), who mostly consulted online social media as well as friends and family; and (3) the Unplugged (*n* = 1,073), who consulted few or no information sources. Compared to the Unplugged, individuals in the other two groups had a higher risk of being concerned (Traditional Media, OR = 2.2; *p* < 0.001; Social Networks and Entourage, OR = 3.1; *p* < 0.001). Communication about pandemic risk should be conveyed based on reliable information and at moderate intervals to safeguard the mental health of individuals.

## Introduction

As of June 2, 2022, a cumulative total of approximately 540 million cases of COVID-19 and 6.3 million deaths had been reported worldwide (1). Within this profound health and social crisis, information sources play an important role in the dissemination of information. In times of crisis, populations receive official communications on how to prepare in advance for the impact of the event, and subsequently, information pertaining to ongoing daily activities is relayed to communities (2). To better understand the epidemic situation, and to protect their health, people have an intense need to seek information from various sources and maintain contact with their community (3, 4). In the context of COVID-19, the high level of fear is probably one of the explanatory factors affecting people's behaviors and perceptions in information seeking (5, 6). Concerns about being infected or transmitting the virus to others were notably visible on social media, where panic seemed to spread faster than the COVID-19 itself (7) On the one hand, information overload phenomenon has led to a lack of confidence in media, especially in social media where a huge amount of untrustworthy content is available (8). On the other hand, inadequate information has sometimes provoked worst consequences in mental health, such as depression, stress and anxiety (9).

Today, this search for information has been facilitated by an explosion of (more or less reliable) information available primarily *via* traditional media (radio/television), new media (web and other digital media), and informal or personal sources of information (blogs, social media, opinion pieces). Nevertheless, this constantly changing and contradictory plethora of news has the potential to affect people's mental health and well-being (10). For example, recent studies have shown that the use of online social networks, *via* negative comments and shares (11), as well as time and frequency of use (12), is associated with significant anxiety. As a result, online social network use often leads to depression, insomnia and consequent emotional problems in individuals (13, 14). Furthermore, Sasaki et al. (15) showed that the use of television to learn about COVID-19 is also a significant factor in consumer anxiety. Other studies have also examined the links between stress, depression, and different information sources in the COVID-19 context (16–20). These have shown that exposure, time spent, and type of information source used are among the factors that trigger negative affect in individuals during COVID-19, generating significant mental health problems.

According to Roy et al. (21), the mental health of individuals, and especially of parents, must be a research priority during the COVID-19 pandemic. As the pandemic continues to ravage the world and protective measures to mitigate its impact are put in place, parents must necessarily expand their use of information sources to learn about COVID-19, as has been empirically demonstrated (22). At the same time, parents are experiencing many disruptions in their daily lives that inevitably augment their anxieties and psychological distress (23, 24), and these may be exacerbated by information sources.

At the same time, the pandemic have also impacted the lives of school-aged children due to restrictions in social interactions with peers and outdoor physical activities during school suspension (25, 26). This vulnerable population may be impacted by deterioration in parent-child relations (27), psychological well-being (28) or violence and maltreatment (29). Moreover, concerns have been raised about the use of digital technologies. School closure during the COVID-19 outbreak has resulted in children spending more time using the Internet (30) which led to many repercussions. For instance, problematic smartphone use contributed to prospective psychological distress among schoolchildren (31, 32) and problematic social media use were associated to depression, anxiety, and stress (33).

In this study, we aimed to understand how COVID-19 related information sources affect parents' concerns of school aged-children in the context of pandemic. This study focused specifically on the concerns of parents of school-aged children, a population that has been relatively understudied to date. We examined existing correlations between information sources (newspapers, online social networks, radio, online forums, friends and family, medical sources), frequency of use (always, often, sometimes, rarely, never), and concerns among parents of school-aged children.

## Materials and methods

### Study design

This study was a secondary analysis of a survey entitled “*Retour des enfants à l'école: intentions des parents d'enfants de Laval en contexte de pandémie (COVID-19).”* (34). That survey targeted parents of children attending schools within the Centre de services scolaires de Laval (Québec). We obtained the necessary approvals from the ethics committee of CHU Sainte-Justine Hospital in Montreal to conduct this study.

### Participants

The survey was conducted using an online questionnaire from August 17 to September 2, 2020. 3,459 parents of school-aged children (4 to 18 years old) from the Centre de services scolaires de Laval have participated in the study. No other clinical inclusion and exclusion criteria was applied to enrollment. The questionnaire was available in French and English.

### Dependent variable

Our dependent variable was a COVID-19 parental concern score, drawn from the iCARE study (available at: https://mbmc-cmcm.ca/covid19/). It was constructed with five questions using a 5-point Likert scale (*very worried, somewhat worried, not very worried, not at all worried, don't know/prefer not to answer*). More specifically, this score aggregates concerns about being infected, about the consequences of an infection, about infecting others and its consequences, and about a new wave of infections in the future. In the end, we obtained a score where the lowest value [0] corresponded to a very low level of concern and the highest value [15] to a high level of concern. By visually inspecting the graphic representations, we dichotomized the concern score into two categories. A split was also applied to this measure in others studies (34, 35).

Moreover, this dependent variable was chosen in two ways. First, it captures both clinical concerns (concerns about being infected and about the consequences of an infection) and psychological concerns (concerns about infecting others and its consequences, and about a new wave of infections in the future thus providing a purposefully broad assessment of concerns experienced by the parents surveyed. Second, previous work on the relationship between concerns has shown that these clinical and psychological concerns has been correlated depending on the type of information source used (16–20). We therefore investigate this point.

### Independent variables

We used questions about how often people consulted several information sources to learn about COVID-19. The proposed information media were radio, online social networks, friends and family, medical sources, online forums, and television. The online social networks suggested to participants were Facebook, Instagram and LinkedIn, while the example of medical sources were physicians or health care professionals. A 5-point Likert scale assessed the frequency with which these media were used to learn about COVID-19 (*never, rarely, sometimes, often, always*). Then, we merged the original five items into two groups (*never/rarely/sometimes*; *often/always*) for ease of analysis. The first group items corresponded to punctual consumers of these media, and the second group items were consistent with regular consumers. We assessed validity of the resulting solution by visually examining the individual composition of clusters (14). We used as control variables the sociodemographic characteristics of individuals, namely age, sex, education level, employment status and immigration status, as they are mostly used to control statistically the outcomes on this subject field (17) and their association with concerns and anxiety (36, 37).

### Data analysis

**Figure 1:**
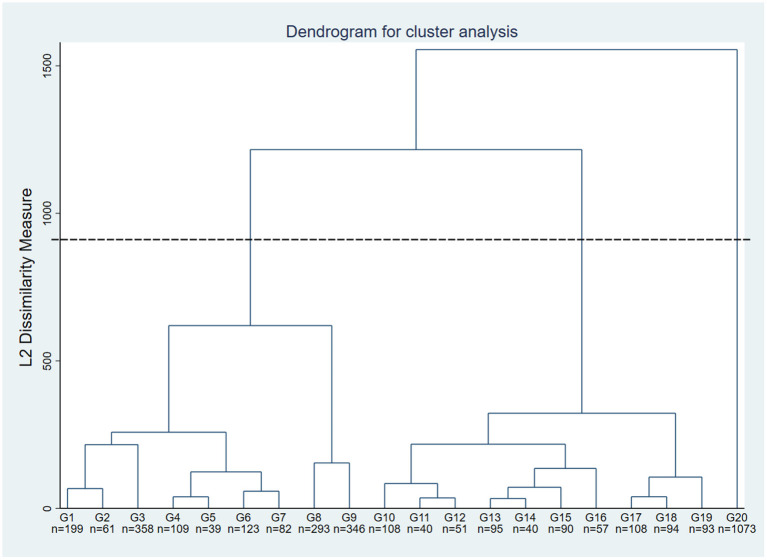
Dendrogram. The sample was initially divided into 20 groups (a partitioning commonly used in the literature) using Stata version 15 software. For illustration purposes, G1 corresponds to Group 1 and *n* = 199 indicates the number of individuals in Group 1, i.e., 199. The black dotted line corresponds to the cutoff point used to determine our three groups minimizing intra-class variance and maximizing inter-class variance.

The different information sources (radio, online social networks, friends and family, medical sources, online forums, television) were the active variables, and the sociodemographic variables (age, sex, education level, employment status, immigration status) were the illustrative variables. The data analysis was conducted in two stages. The first stage consisted of constructing a taxonomy of respondents according to the sources of information consulted, based on a multiple correspondence analysis (MCA) combined with a hierarchical ascending classification (38, 39). MCA is a factor analysis method suited to the analysis of categorical variables. The MCA revealed the most significant data structures (factorial axes). The number of axes retained from the MCA was determined using the elbow criterion. In keeping with the general philosophy of multiple factor analysis, we did not use axis rotation, since the MCA is only an intermediate step toward developing a taxonomy (40). Using the axes retained from the MCA, we performed a hierarchical ascending classification using Ward's algorithm (41). This proven technique for creating homogeneous groups was used to avoid the creation of chain effects that could prevent us from obtaining clear distinctions between groups. The objective was to group individuals according to their characteristics by minimizing intra-class variance and maximizing extra-class variance to form homogeneous groups. We chose the optimal number of classes by examining the Euclidean distances (L2 dissimilarity measure) of the dendrogram (Figure 1).

The second stage consisted of an analysis to examine the association between the taxonomy profiles and parental concern. We performed an ANOVA analysis with a Bonferroni correction to compare statistically the concern means of the information groups, and we used a binomial logistic regression to explain individuals' concern levels. The groups from the hierarchical ascending classification were included as the explanatory variable of interest in the logistic model, and the sociodemographic variables were the control variables.

## Results

Table 1 presents the numbers (*n*) and percentages (%) of parents who participated in the study according to certain sociodemographic characteristics.

**Table 1:** Sociodemographic statistics.

		***n***	**%**	**CI (95%)**
**Sex**	Male	718	20.8	[19.4; 22.1]
	Female	2,741	79.2	[77.9; 80.6]
**Age**	Under 35 years	282	8.2	[7.3; 9.1]
	35–44 years	2,059	59.5	[57.9; 61.1]
	45 years +	1,118	32.3	[30.8; 33.9]
**Education level**	High school diploma or less	522	15.0	[13.9; 16.3]
	College diploma	668	19.3	[18.0; 20.7]
	University degree	2,269	65.6	[64.0; 67.1]
**Employment status**	Employed	515	85.1	[83.9; 86.3]
	Unemployed	2,944	14.9	[14.9; 16.1]
**Immigration status**	Born in Canada	2,138	61.8	[60.2; 63.4]
	Immigration > 10 years	970	28.0	[26.6; 29.6]
	Immigration between 5 and 9 years	206	6.0	[5.2; 6.8]
	Immigration < 5 years	145	4.2	[3.6; 4.9]
	Total	3,459	100	

### Multiple correspondence analysis and hierarchical ascending classification

In the first stage of the analysis we performed an MCA. After observing the eigenvalues and appropriate modalities for the axes, we chose to retain two dimensions using the elbow criterion, thereby synthesizing 94.22% of the main inertia. Adding an incremental axis did not increase this explained inertia. In the second stage, we used the two axes to perform a hierarchical ascending classification. Euclidean distance analysis of the dendrogram (Figure 1) suggested partitioning our sample into three groups. These groups minimized intra-class variance and maximized inter-class variance.

The first group (*n* = 1,610), which we called Traditional Media, consisted of individuals who reported often or always seeking information about COVID-19 *via* traditional sources of information such as newspapers, television, or radio. The second group (*n* = 776), called Online Social Networks and Entourage, consisted of individuals who reported often or always seeking information about COVID-19 *via* online social networks, online forums, or friends and family. The third group, called Unplugged (*n* = 1,073), was composed of individuals who never or rarely consulted any of the information sources put forward in the questionnaire to learn about COVID-19. All descriptive statistics are presented in Table 2.

**Table 2:** Distribution of information sources by groups and sociodemographic characteristics.

	**Initial sample** **(*N* = 3,459)**	**Traditional media** **(*n* = 1,610)**	**Online social networks and entourage** **(*n* = 776)**	**Unplugged** **(*n* = 1,073)**
	***N***	***n (%)***	***n (%)***	***n (%)***
**Information sources**				
**Television**				
Never/Rarely/Sometimes	2,200	780 (35.4)	347 (15.8)	1,073 (48.8)
Often/Always	1,259	830 (65.9)	429 (34.1)	0 (0.0)
**Newspapers**				
Never/Rarely/Sometimes	1,938	445 (23.0)	420 (21.6)	1,073 (55.4)
Often–Always	1,521	1,165 (76.6)	356 (23.4)	0 (0.0)
**Online social networks**				
Never/Rarely/Sometimes	2,729	1,453 (53.2)	203 (7.5)	1,073 (39.3)
Often–Always	730	157 (21.5)	573 (78.5)	0 (0.0)
**Radio**				
Never/Rarely/Sometimes	2,769	1,185 (42.8)	511 (18.5)	1,073 (38.8)
Often–Always	690	425 (61.6)	265 (38.4)	0 (0.0)
**Online forums**				
Never/Rarely/Sometimes	3,247	1,610 (49.6)	564 (17.4)	1,073 (33.0)
Often–Always	212	0 (0)	212 (100.0)	0 (0.0)
**Friends and family**				
Never/Rarely/Sometimes	2,790	1,496 (53.6)	221 (7.9)	1,073 (38.5)
Often–Always	669	114 (17.0)	555 (83.0)	0 (0.0)
**Medical sources**				
Never/Rarely/Sometimes	2,484	941 (37.9)	470 (18.9)	1,073 (43.2)
Often–Always	975	669 (68.6)	306 (31.4)	0 (0.0)
**Sociodemographics**				
**Sex**				
Male	718	343 (47.8)	154 (21.4)	221 (30.8)
Female	2,741	1,267 (46.2)	622 (22.7)	852 (31.1)
**Age**				
Under 35 years	282	93 (33.0)	76 (27.0)	113 (40.0)
35–44 years	2,059	965 (46.9)	444 (21.6)	650 (31.5)
45 years +	1,118	552 (49.4)	256 (22.9)	310 (27.7)
**Education**				
High school or less	522	183 (35.0)	156 (30.0)	183 (35.0)
College	668	264 (39.5)	144 (21.6)	260 (38.9)
University	2,269	1,163 (51.2)	476 (21.0)	630 (27.8)
**Employment**				
Unemployed	515	193 (37.5)	186 (36.0)	136 (26.5)
Employed	2,944	1,417 (48.2)	590 (20.0)	937 (31.8)
**Immigration**				
Born in Canada	2,138	1,095 (51.2)	297 (13.9)	746 (34.9)
Immigration 10 years or more	970	406 (41.8)	310 (32.0)	254 (26.2)
Immigration 5–9 years	206	66 (32.0)	88 (42.7)	52 (25.3)
Immigration < 5 years	145	43 (29.7)	81 (55.9)	21 (14.5)
**Concern score**				
Mildly concerned (0–9)	1,579	694 (44.0)	227 (14.4)	658 (41.6)
Strongly concerned (10–15)	1,880	916 (48.7)	549 (29.2)	415 (22.1)

### Sociodemographic statistics

From a sociodemographic standpoint (Table 2), we note that nearly 50% of individuals aged 35 years and over belonged to the Traditional Media group. Among those under 35 years, 40% belonged to the Unplugged group. As well, 51.3% of university graduates were in the Traditional Media group, whereas this percentage dropped to 21% for the Online Social Networks and Entourage group. Finally, among Canadian-born individuals, 51.2% were in the Traditional Media group, while only 13.9% were in the Online Social Networks and Entourage group. More specifically, we note that this last group was composed largely of individuals who had immigrated to Canada within the past 10 years. All the sociodemographic statistics are presented in Table 2.

### Statistics of the dependent variable

The mean concern score in our sample was 9.5/15 (s.d. = 3.87). We calculated a mean for each profile in the taxonomy. The Online Social Networks and Entourage group had a mean score of 11.0, making it the most concerned group. The last group, the Unplugged, had the lowest mean, at 8.0 (Table 3). The score for the middle group, Traditional Media, was 9.8. Analysis of variance with the Bonferroni correction showed the differences in means between each pair of groups to be significant at the 1% level (Table 3).

**Table 3:** Mean level of concern and analysis of variance (Bonferroni correction).

	**Initial sample** **(*N* = 3,459)**	**Traditional media** **(*n* = 1,610)**	**Online social networks and entourage** **(*****n*** = **776)**		**Unplugged** **(*n* = 1,073)**
**Mean concern score (s.d)**	9.51 (3.87)	9.79 (3.63)	11.01 (3.72)		8.01 (3.81)
	**Sum of squares**	**Degrees of freedom**	**Means of squares**	**Fisher stat**	**Prob** > **F**
Between	4,284.64	2	2,142.32	155.85	*<0.001*
Within	47,506.91	3,456	13.75		
Total	51,791.55	3,458	14.97		
*Bartlett's test for equality of variances: chi^2^ =2.96; Prob>chi^2^ = 0.228*					
**Bonferroni multiple comparison test**					
		**Traditional media**		**Online social networks and entourage**	
**Online Social Networks and Entourage**		1.21			
*P-value*		*<0.001*			
**Unplugged**		−1.78		−2.9	
*P-value*		*<0.001*		*<0.001*	

### Binomial logistic regression

**Figure 2:**
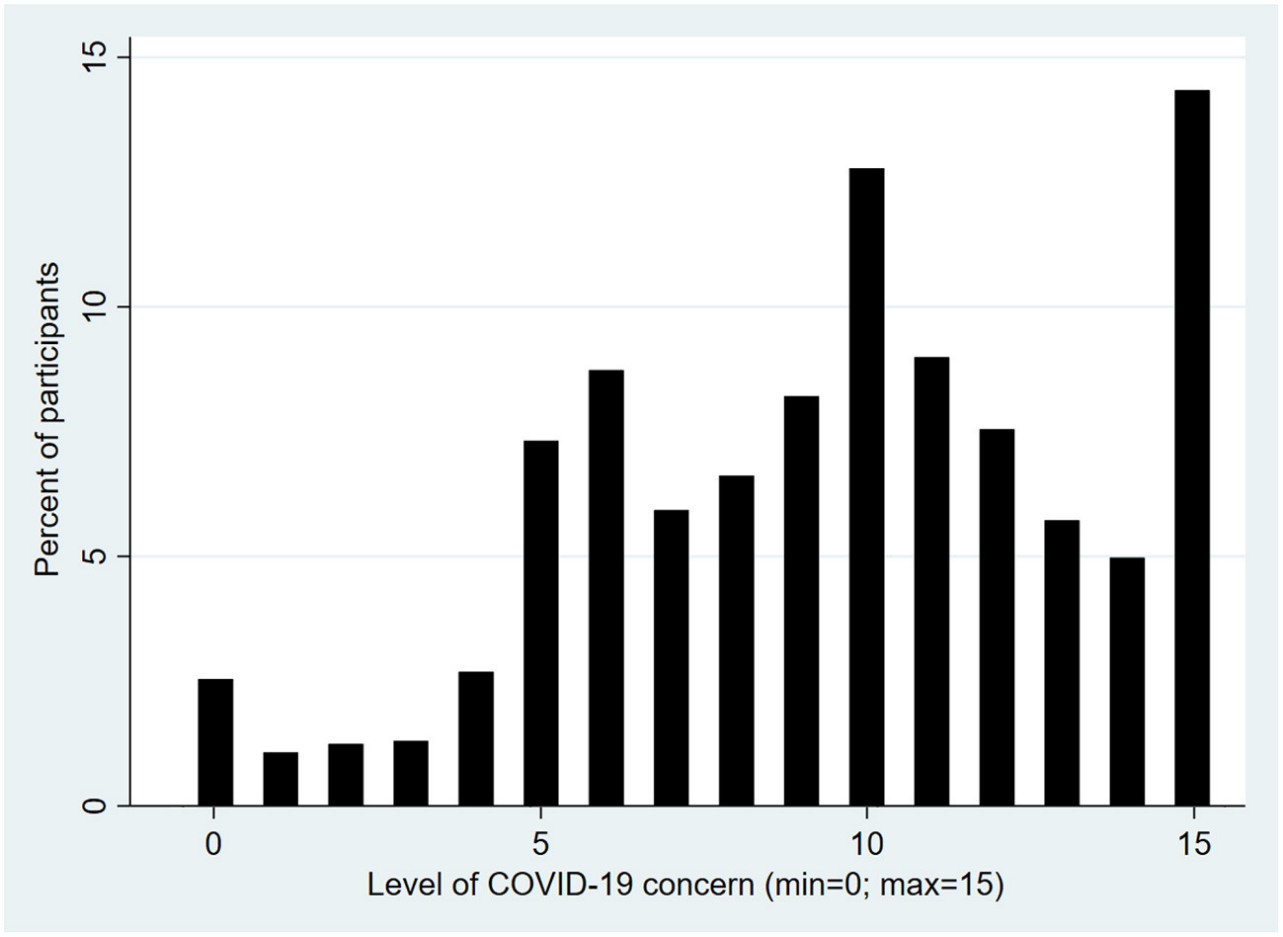
Distribution of level of COVID-19 concern.

We used binomial logistic regression (Table 4) to analyze the association between groups and parents' level of concern. As described in the Materials and methods section, the concern score was separated into two categories to obtain our dependent variable. The distribution of level of COVID-19 concern is available in Figure 2. Individuals with a score of 0 to 9 inclusive were considered to have a lower level of concern (*n* = 1,579, 45.6%), and those with a score of 10 to 15 inclusive were considered more concerned (*n* = 1,880, 54.4%).In the Traditional Media group, the probability of being among the most concerned (scoring 10 to 15) was multiplied by a factor of 2.2 compared to the Unplugged group (OR = 2.2; *p* < 0.001). In the Online Social Networks and Entourage group, the probability of being among the most concerned is multiplied by a factor of 3.1 compared to the Unplugged group (OR = 3.1; *p* < 0.001).

**Table 4:** Binomial logistic regression.

				**N**	**3,459**
				**Pseudo R** ^2^	**0.0664**
**Dependent variable: Concern score**					
			**OR**	***p***	**CI (95%)**
Variable of interest	**Group** (ref: Unplugged)	Traditional media	2.21	<0.001	[1.88; 2.60]
		Online social networks and entourage	3.18	<0.001	[2.59; 3.92]
Sociodemographic variables	**Sex of parents** (ref: Male)	Female	1.31	0.002	[1.10; 1.57]
	**Age** (ref: Under 35 years)	35–44 years	1.12	0.412	[0.85; 1.47]
		45 years +	1.09	0.530	[0.82; 1.46]
	**Employment status** (ref: Unemployed)	Employed	0.69	<0.001	[0.53; 0.83]
	**Education** (ref: High school diploma or less)	College	0.87	0.286	[0.68; 1.11]
		University	0.69	0.001	[0.56; 0.85]
	**Immigration status** (ref: Born in Canada)	Immigration 10 years+	2.01	<0.001	[1.71; 2.39]
		Immigration 5–9 years	1.63	0.002	[1.20; 2.23]
		Immigration <5 years	1.65	0.010	[1.13; 2.43]

Among the control variables, employed individuals were 1.5 times less likely than unemployed individuals to be among the most concerned (OR = 0.69, or 1/0.69 = 1.45; *p* < 0.001). Regarding immigration, we note that individuals born outside of Canada were more likely to be among the concerned than those born in Canada. For example, individuals who had immigrated to Canada in the past 5 years were 1.7 times more likely to be among the concerned persons than those born in Canada.

## Discussion

The objective of this study was to analyze whether information sources and their frequency of use were associated with levels of concern among parents of school-aged children seeking information about COVID-19. Our study showed that, in our sample, parents who frequently used online social networks, online forums, and friends and family to obtain information about COVID-19 had a higher level of concern than those who never sought information about the pandemic.

### Online social networks and strong concerns

In the literature, exposure to online social networks is widely associated with significant concern (10, 19, 42), which is consistent with the results of our study. The causes of this association between concern and online social networks have been the subject of several studies. One hypothesis is that online social networks are the source of numerous publications of all kinds that are not always scientifically sound. If the content has frightening information or graphics (in image or video format), this may exacerbate individuals' distress levels (43). As Cinelli et al. (44) showed empirically, these platforms are also often prone to slander, false rumors, and dubious information, especially in the context of COVID-19. This onslaught of information, sometimes spreading faster than the virus itself, creates uncertainty and concern (45, 46) and generates a certain distrust among online social network consumers, who then comply only partially with health restrictions (47). For example, users of some online social networks have been found to have lower levels of compliance with social distancing compared to users of traditional media (48).

With respect to frequency of use, the study by Shensa et al. (14) also used a cluster analysis. From a nationally representative sample of 1,730 US adults, they identified five clusters based on online social network usage, which they labeled Wired, Connected, Diffuse Dabblers, Concentrated Dabblers, and Unplugged. The authors showed that membership in the Wired and Connected clusters increased the likelihood of elevated symptoms of depression and anxiety. The main reason was that, in a crisis context, online social networks have significant informational and social potential and are more often used by parents with higher levels of anxiety in crisis situations (22, 49). Thus, the analyses conducted in our sample were not due to chance and appear to corroborate the findings of the existing literature on the subject.

### Traditional media and concern

In our sample, the Traditional Media group using primarily television also exhibited a high level of concern, but to a lesser extent. This result is consistent with the work of Sasaki et al. (15) showing that the use of television to learn about COVID-19 was positively associated with concern, but on a lesser scale than for online social networks. When television channels regularly report news about hospitals and overwhelmed health professionals during peak epidemic periods, people's fears and concerns about COVID-19 are amplified (16). However, the engagement of vulnerable people in coronavirus-related preventive behaviors is significantly associated with impediments, benefits, self-efficacy, and trust in television for information about COVID-19 (50).

Thus, there exists a certain ambiguity between the concern mediated by images on television and that medium's reliable informational power, such that it continues to be one of the most consulted sources of information when it comes to learning about an epidemic context such as COVID-19.

### The unplugged and attenuated concerns

An important finding in our analysis concerned the Unplugged information group. In our sample, 1,073 of 3,459 adults responded *never, rarely*, or *sometimes* with regard to learning about COVID-19 through the media put forward in the questionnaire. The results from the analyses of variances (Bonferroni correction in Table 3 and binomial logistic regression in Table 4) indicated that these adults were statistically less concerned than the other groups. However, few studies have shown that moderation in media consumption may not be associated with mental health risks for adults (51). In the study mentioned earlier, Shensa et al. (14) showed that membership in the Wired and Connected clusters increased the likelihood of elevated symptoms of depression and anxiety. The other three clusters were not statistically associated with depression and anxiety.

### Limitations

Our results should, however, be interpreted while keeping in mind certain limitations. From a methodological point of view, the study could not establish a causal relationship between media exposure and concern, due to the cross-sectional design. As the epidemic continues, it is possible that media use may escalate subsequent negative psychological outcomes, which in turn would encourage increased media use (10). Second, our study focused only on investigating the effects of certain media on parental concern without addressing other components of mental health. For example, other studies have shown that high exposure to social media during a natural crisis such as COVID-19 also has an impact on depression, emotions, and posttraumatic stress disorder (16–18, 52). Third, because media use was self-reported, the measures may suffer from social desirability and/or recall bias. Fourth, since our sample was limited to 3,459 parents of school-aged children in Laval, Quebec, it is not certain that the results can be generalized to samples from other countries. Plus, more female (79.2%) than male (20.8%) participated in this study. Fifth, the binomial regression model has a predictive rate of approximately 63%, accurately classifying 2,166 individuals out of the 3,459 total observations. The area under the receiver operating characteristic curve (AUROC) is equal to 0.67. Our model has a correct discrimination power but could be improved by integrating other mental health variables (stress, depression, anxiety, etc.).

From a theoretical point of view, the information seeking variable failed to distinguish whether parents were more likely to consume valuable health-related information or misinformation. Although this was not the objective of this study, this information is important because misinformation has been found to worsen mental health consequences (9).

## Conclusion

The results are consistent with previous studies demonstrating the association between the use and frequency of information sources consulted and mental health. This study sheds new light by focusing specifically on the concerns of parents of school-aged children, a population that has been relatively understudied to date. In our sample, adults who often or always used online social networks, friends and family, and television to learn about COVID-19 had higher levels of concern compared to parents who never, rarely, or sometimes used information sources.

Our findings suggest time spent using online social networks during the initial phase of an outbreak should be limited to reduce exposure to stressful and possible unreliable content. Public health authorities should promote information messages on all communication channels in order to reach a wide coverage of the population, while notifying moderately the population with reliable and verified information. This could directly reduce negative psychological consequences for parents and indirectly for children. Particular attention should then be focused on online social networks and television, as these are widely used by the population. Further research, covering the different phases over the course of the pandemic, could provide a better assessment of parental concern by linking it with the sources of the information they consume over the long term.

## Data availability statement

The raw data supporting the conclusions of this article will be made available by the authors, without undue reservation.

## Ethics statement

The Ethics Committee of the CHU Sainte-Justine in Montreal approved the study (#2021-3032). The patients/participants provided their written informed consent to participate in this study.

## Author contributions

YA, OD, and RBDS contributed to the conception and design of the study and revised the article critically. YA and RBDS contributed to the analysis and interpretation of data. YA and OD drafted the article. All authors read and approved the final manuscript.

## Funding

This study was supported by a grant from the Centre interuniversitaire de recherche en analyse des organisations (CIRANO) and from the Fonds de recherche du Québec–Santé (OD is an FRQS Junior 1 clinical research scholar).

## Conflict of interest

The authors declare that the research was conducted in the absence of any commercial or financial relationships that could be construed as a potential conflict of interest.

## Publisher's note

All claims expressed in this article are solely those of the authors and do not necessarily represent those of their affiliated organizations, or those of the publisher, the editors and the reviewers. Any product that may be evaluated in this article, or claim that may be made by its manufacturer, is not guaranteed or endorsed by the publisher.
